# Association of per- and polyfluoroalkylated substances/heavy metals and bone health in children and adolescents

**DOI:** 10.3389/fpubh.2024.1431001

**Published:** 2024-10-10

**Authors:** Yumeng Wei, Yuxiao Zhang, Qiaoyun Ji, Sufei Yang, Fan Yang

**Affiliations:** ^1^Department of Pediatrics, West China Second University Hospital, Sichuan University, Chengdu, China; ^2^Key Laboratory of Birth Defects and Related Diseases of Women and Children (Sichuan University), Ministry of Education, Chengdu, China

**Keywords:** bone mineral density, children, adolescents, PFASs, heavy metals

## Abstract

**Background:**

Research on the correlation between exposure to per- and polyfluoroalkylated substances (PFASs)/heavy metals and bone health during childhood and adolescence is limited. Considering their role as endocrine disruptors, we examined relationships of six PFASs and three heavy metals with bone mineral density (BMD) in children and adolescents using representative samples from the National Health and Nutrition Examination Survey (NHANES).

**Methods:**

The study included 622 participants aged 12–19. The relationship between single pollutant and lumbar spine and total BMD was studied using linear regression analyses. Additionally, Bayesian Kernel Machine Regression (BKMR) models were applied to assess the joint effects of multiple PFASs and heavy metals exposure on the lumbar spine and total BMD.

**Results:**

Statistically significant differences were noted in the serum concentrations of perfluorooctanoic acid (PFOA), perfluorooctane sulfonic acid (PFOS), perfluorohexane sulfonic acid (PFHxS), blood lead (Pb), and blood manganese (Mn) between male and female participants (all *p* < 0.05). Single-exposure studies have shown that Mn was negatively correlated with lumbar spine BMD and total BMD. Multivariate linear regression models revealed that, in the male group, total bone density decreased as the blood PFOA levels [95% CI = (−0.031, −0.001), *p* = 0.040] and blood manganese levels [95% CI = (−0.009, −0.002), *p* = 0.004] increased. Similarly, lumbar spine bone density decreased as the blood manganese levels [95% CI = (−0.011, −0.002), *p* = 0.009] increased. In the female group, total bone density decreased as the serum PFNA levels [95% CI = (−0.039, 0.000), *p* = 0.048] increased. As shown in the BKMR model, the joint effects of pollutant mixtures, including Mn, were negatively associated with both the lumbar spine and total BMD. Among the pollutants analyzed, Mn appeared to be the primary contributor to this negative association.

**Conclusion:**

This study suggests that exposure to certain PFASs and heavy metals may be associated with poor bone health. Childhood and adolescence are crucial stages for bone development, and improper exposure to PFASs and heavy metals during these stages could potentially jeopardize future bone health, consequently raising the risk of osteoporosis in adulthood.

## 1 Introduction

The process of bone development encompasses both linear growth and the accumulation of bone mass. In childhood, bone growth is primarily characterized by linear growth, with an average growth rate of 5–6 cm/year; during puberty, the emphasis shifts toward the accumulation of bone mass (1), achieving 40–60% of adult bone mass during adolescence, with 90% of peak bone mass (PBM) accumulated by the age of 18 (2). As reported, a 10% increase in PBM can postpone osteoporosis onset by 13 years, while a 6.4% reduction during childhood doubles the risk of fractures in adulthood (3). Maintaining optimal bone health and fostering longitudinal growth throughout childhood and adolescence can prevent fractures related to osteoporosis in adulthood (4). Hence, maintaining bone health during childhood and adolescence is imperative for mitigating the risk of osteoporosis-related diseases and fractures in adulthood.

Endocrine disrupting chemicals (EDCs) are a class of compounds that can confuse the hormonal system in the human body (5). By mimicking the function of hormones, they disrupt the body’s metabolic processes and hormonal balance. Bone, as an active connective tissue and endocrine organ, is particularly sensitive to EDCs (6). These chemicals can have harmful effects on bone tissue by altering bone modeling and remodeling processes, as well as altering the release of hormones, cytokines, chemokines, and growth factors throughout the body (7). Certain PFASs and heavy metals have been classified as EDCs and are being monitored in human biomonitoring studies (8, 9). Human exposure to PFASs can occur through various pathways, including diet, drinking water, or dust (10, 11), with dietary intake and migration from food packaging or cookware being the main exposure pathways (12). A smaller portion of exposure may arise from indoor environments, specifically dust and air (13). PFOA and PFOS are the most common PFASs in the environment, but with their phase-out, children and adolescents in the United States have been observed to have high exposure rates to certain PFASs (14), such as PFNA, PFDA, PFHxS, and PFUA. Optimal concentrations of specific metals such as zinc, iron, and copper in the body are essential for maintaining normal physiological functions (15, 16). However, exposure to heavy metals as environmental pollutants can induce genes alterations and increase susceptibility to disease (17). Existing evidence suggests that excessive exposure to metals such as cadmium, lead, manganese may affect bone health (18). Children may be exposed to excessive cadmium, manganese, and lead through contact with contaminated freshwater, soil, air, food, and smoking (19–21). Given that children are undergoing critical stages of growth and development, their susceptibility to environmental pollutants warrants careful consideration. It is essential to examine the potential impact of PFASs and heavy metals on bone health in pediatric and adolescent populations. To delve deeper into this potential impact, this study gathered pertinent data from the National Health and Nutrition Examination Survey (NHANES) spanning 2011 to 2018 to investigate the relationship between blood PFASs, heavy metals, and bone mineral density (BMD) among individuals aged 12–19 years.

## 2 Methods

### 2.1 Study population

National Health and Nutrition Examination Survey constitutes a series of cross-sectional surveys conducted by the Centers for Disease Control and Prevention (CDC) in the United States. These surveys aim to focus on the health and nutrition of American adults and children. Four NHANES cycles (2011–2012, 2013–2014, 2015–2016, and 2017–2018) were selected in this study. Data from the NHANES database (2011–2018) for 5,215 participants aged between 12 and 19 years were extracted. Subsequently, participants who lacked BMD data (*n* = 1,098), serum PFAS concentration data (*n* = 2,968), and blood heavy metal concentration data (*n* = 460), as well as those with incomplete covariate information (*n* = 67) were excluded. A total of 622 participants aged between 12 and 19 were ultimately included in this study.

### 2.2 Measurements of serum PFAS and blood levels of metals

Serum concentrations of six PFASs were studied: perfluorooctanoic acid (PFOA), perfluorooctane sulfonic acid (PFOS), perfluorononanoic acid (PFNA), perfluoroundecanoic acid (PFUA), perfluorodecanoic acid (PFDA), and perfluorohexane sulfonic acid (PFHxS). After collection, the samples were refrigerated and stored in −20°C polypropylene or polyethylene containers (22). The serum concentrations of PFASs in the samples were analyzed using solid phase extraction-high performance liquid chromatography-turbo ion spray-tandem mass spectrometry (SPE-HPLC-TIS-MS/MS) (23). The serum concentrations of the three heavy metals: lead (Pb), cadmium (Cd), and manganese (Mn), were determined via inductively coupled plasma dynamic reaction cell mass spectrometry (ICP-DRC-MS) (24). Concentrations below the limit of detection (LOD) were replaced by the LOD divided by √2 (25). About 100% of PFOA, PFOS, PFHxS, and Mn observations were above the LOD. For Pb, 98% of observations were above the LOD. Similarly, 97% of PFNA and 75.7% of PFDA observations were above the LOD, while 62.4% of Cd and 37.1% of PFUA observations were above the LOD. Detailed information on the analytical methods and quality assurance/quality control procedures is available at https://www.cdc.gov/nchs/nhanes/index.htm.

### 2.3 Bone mineral density

The International Society for Clinical Densitometry recommends the lumbar spine as the skeletal site for pediatric bone density measurement (26), and total BMD measurement is applicable to children aged 3 years and older (27). Therefore, lumbar spine BMD and total BMD were selected as the dependent variables for our analysis. Data on lumbar spine BMD and total BMD for participants aged over 8 years were derived from the DXXLSA dataset. BMD was assessed using dual-energy X-ray absorptiometry (DXA) using a Hologic Discovery A bone densitometer and Apex v3.2 (28). The accuracy of the scans was upheld through routine quality control procedures and meticulous reviews conducted by experts for all scans. More details on the data collection are available at: https://wwwn.cdc.gov/Nchs/Nhanes/2011-2012/DXX_G.XPT, https://wwwn.cdc.gov/Nchs/Nhanes/2013-2014/DXX_H.XPT, https://wwwn.cdc.gov/Nchs/Nhanes/2015-2016/DXX_I.XPT, and https://wwwn.cdc.gov/Nchs/Nhanes/2017-2018/DXX_J.XPT.

### 2.4 Other covariates

Covariates considered in this study encompassed race, sex, age, poverty income ratio (PIR), body mass index (BMI), and serum 25-Hydroxyvitamin-D (25(OH)D) concentration. Active and passive smoking have been confirmed to reduce BMD levels. Cotinine, the primary metabolite of nicotine, is a reliable biomarker for assessing exposure to both active and passive smoking. A higher PIR reflects better socioeconomic status and household income. Data on covariates were collected through home interviews, physical examinations, laboratory measurements, and questionnaires. More details about the data collection are available at NHANES—National Health and Nutrition Examination Survey Homepage.^1^

### 2.5 Statistical analysis

Statistical analyses were conducted utilizing SPSS v26, R v4.4.1 and GraphPad Prism v9.5. Population characteristics, exposures, and outcomes were described using Mean ± standard error (SE) or percentage, and differences between male and female participants were compared using a *t*-test or chi-square test. The linear regression model was applied to assess the individual effect of each heavy metal and PFAS exposure on BMD adjusting for all mentioned covariates. The multiple-metal linear regression model was also constructed, including all six PFASs and three studied heavy metals. Additionally, Bayesian kernel machine regression (BKMR) models were applied to assess the combined effects of mixed heavy metal and PFAS exposure on BMD. Results were reported as regression coefficients and the corresponding 95% confidence interval (CI). A *p* value of less than 0.05 was deemed statistically significant.

## 3 Results

### 3.1 Basic information

This study included a total of 622 participants, with an average age of 15.56 ± 2.26 years. Of these participants, 52.3% were males and 47.7% were females. The number of individuals aged 12–15 and 16–19 each accounted for 49.4 and 50.6%, respectively. Mexican American, Non-Hispanic Blacks, and Non-Hispanic Whites constituted 19.9, 21.5, and 30.4%, respectively. The average BMI was 24.50 ± 6.31 kg/m^2^, with those who were normal weight or underweight, overweight, and obese constituting 60.0, 16.7, and 23.3%, respectively. The average PIR was 2.07 ± 1.52; the average total BMD was 1.040 ± 0.119 g/cm^2^, and the average lumbar spine BMD was 0.978 ± 0.150 g/cm^2^, respectively.

No significant differences were noted in age, race, BMI, PIR, serum 25(OH)D concentration, and serum cotinine concentration between the male and female groups (all *p* > 0.05). However, statistically significant differences in total BMD and lumbar spine BMD (all *p* < 0.05) were observed. The total BMD of the male group was significantly higher than that of the female group (1.062 ± 0.133 vs. 1.016 ± 0.095) (*p* < 0.05), while the lumbar spine BMD of the male group was significantly lower than that of the female group (0.959 ± 0.165 vs. 0.998 ± 0.129) (*p* < 0.05), as shown in Table 1.

**Table 1:** Basic Information of study population.

Variable	Study population	Male	Female	χ^2^/*t*	*p*
Number of participants	622	325	297		
Age (years)					
*x-±s*	15.56 ± 2.26	15.71 ± 2.25	15.39 ± 2.26	1.735	0.083
12–15 (years)	307 (49.4%)	154 (47.4%)	153 (51.5%)	1.059	0.303
16–19 (years)	315 (50.6%)	171 (52.6%)	144 (48.5%)		
Race					
Mexican American	124 (19.9%)	65 (20.0%)	59 (19.9%)	3.160	0.532
Other Hispanic	56 (9.0%)	26 (8.0%)	30 (10.1%)		
Non-Hispanic Blacks	133 (21.5%)	77 (23.7%)	57 (19.2%)		
Non-Hispanic Whites	189 (30.4%)	100 (30.7%)	89 (30.0%)		
Other races	119 (19.1%)	57(17.6%)	62 (20.8%)		
BMI (kg/m^2^)					
*x-±s*	24.50 ± 6.31	24.87 ± 6.21	24.08 ± 6.41	1.565	0.118
Normal weight or underweight	373 (60.0%)	186 (57.2%)	187 (63.0%)	2.471	0.480
Overweight	104 (16.7%)	56 (17.2%)	48 (16.2%)		
Obesity	145 (23.3%)	83 (25.6%)	62 (20.8%)		
PIR					
*x-±s*	2.07 ± 1.52	2.07 ± 1.46	2.07 ± 1.60	0.056	0.956
25(OH)D (nmol/L)					
*x-±s*	55.69 ± 20.82	56.60 ± 19.81	54.70 ± 21.87	1.135	0.257
Serum cotinine (ng/mL)					
*x-±s*	14.37 ± 64.15	17.30 ± 65.21	11.14 ± 56.27	1.256	0.210
Total BMD (g/cm^2^)					
*x-±s*	1.040 ± 0.119	1.062 ± 0.133	1.016 ± 0.095	4.957	< 0.001
Lumbar spine BMD (g/cm^2^)					
*x-±s*	0.978 ± 0.150	0.959 ± 0.165	0.998 ± 0.129	−3.278	0.001

### 3.2 PFAS and metal levels in study population

The PFOA, PFOS, PFNA, PFUA, PFDA, and PFHxS concentrations in the study subjects were 1.630 ± 0.935 ng/mL, 4.117 ± 3.087 ng/mL, 0.698 ± 0.557 ng/mL, 0.115 ± 0.103 ng/mL, 0.176 ± 0.155 ng/mL, and 1.584 ± 2.402 ng/mL, respectively. The concentrations of cadmium, lead, and manganese were 0.210 ± 0.256 μg/L, 0.581 ± 0.429 μg/dL, and 11.091 ± 3.741 μg/L, respectively.

**Figure 1:**
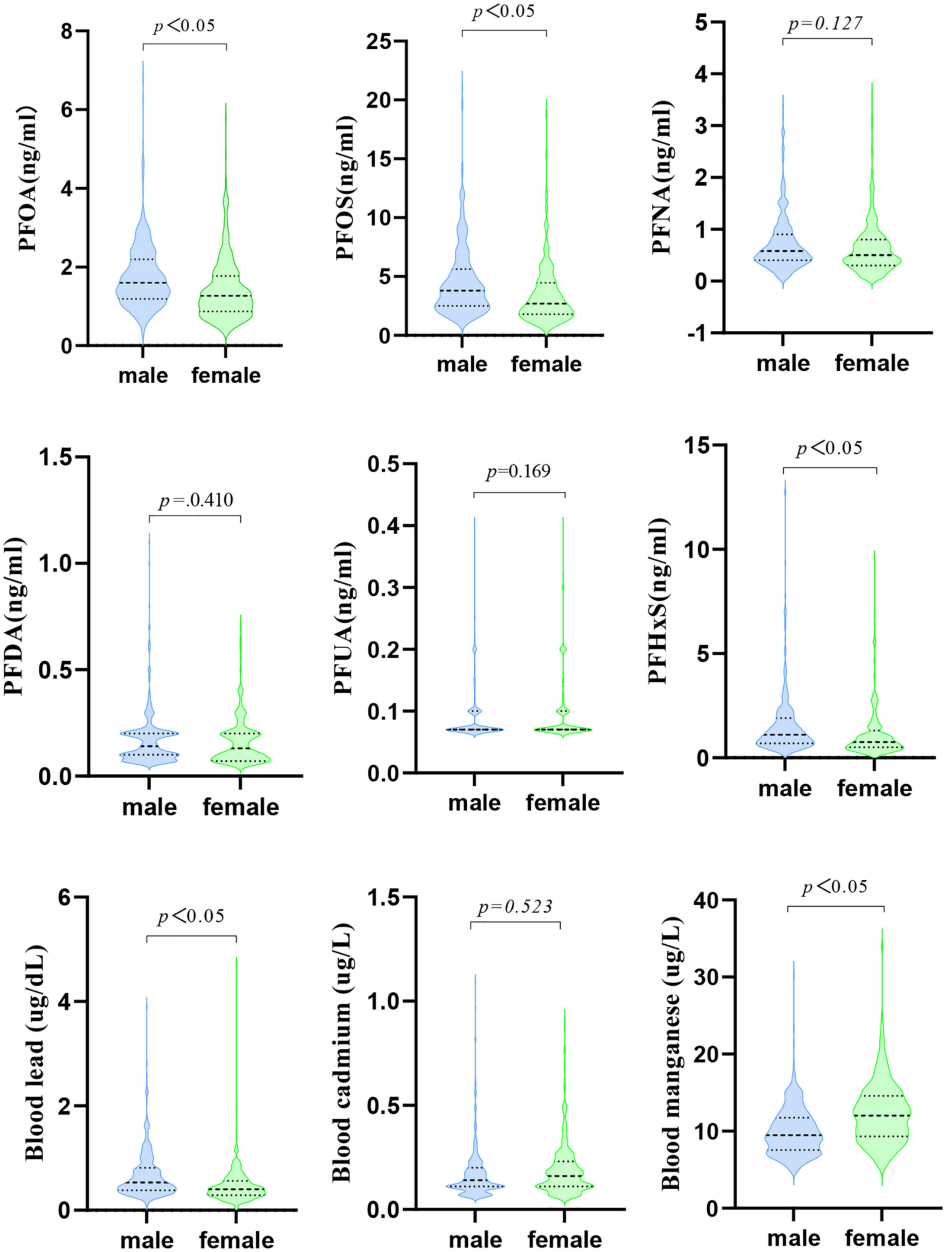
PFAS and metal levels in study population.

A univariate analysis of PFAS and metal levels in both male and female groups was conducted to explore the differences in PFAS and metal levels between the sexes. The results indicated statistically significant differences in the levels of PFOA, PFOS, PFHxS, blood lead, and blood manganese between male and female participants (*p* < 0.05). The concentrations of PFOA, PFOS, and PFHxS (1.802 vs. 1.441, 4.654 vs. 3.529, and 1.816 vs. 1.330), as well as blood lead levels (0.681 vs. 0.471), were higher in males, while blood manganese levels (12.310 vs. 9.978) were higher in females, as illustrated in Table 2 and Figure 1.

**Table 2:** PFAS and metal levels in study population.

Variable	Study population	Male	Female	*t*	*p*
PFOA (ng/mL)	1.630 ± 0.935	1.802 ± 1.001	1.441 ± 0.811	4.896	< 0.001
PFOS (ng/mL)	4.117 ± 3.087	4.654 ± 3.270	3.529 ± 2.762	4.613	< 0.001
PFNA (ng/mL)	0.698 ± 0.557	0.730 ± 0.556	0.662 ± 0.557	1.529	0.127
PFUA (ng/mL)	0.115 ± 0.103	0.110 ± 0.090	0.121 ± 0.115	−1.377	0.169
PFDA (ng/mL)	0.176 ± 0.155	0.181 ± 0.171	0.171 ± 0.135	0.824	0.410
PFHxS (ng/mL)	1.584 ± 2.402	1.816 ± 2.533	1.330 ± 2.228	2.529	0.012
Cadmium (μg/L)	0.210 ± 0.256	0.217 ± 0.317	0.203 ± 0.166	0.640	0.523
Lead (μg/dL)	0.581 ± 0.429	0.681 ± 0.468	0.471 ± 0.352	6.269	< 0.001
Manganese (μg/L)	11.091 ± 3.741	9.978 ± 3.166	12.310 ± 3.941	−8.168	< 0.001

### 3.3 Linear regression analysis

The individual effects of each of the heavy metals on BMD, as assessed using the linear regression model, are provided in Table 3. After adjusting for potential confounders, Mn was found to be negatively associated with both lumbar spine BMD [*β* = −0.003, 95% CI = (−0.007, −0.001), *p* < 0.05] and total BMD [*β* = −0.004, 95% CI = (−0.005, −0.001), *p* < 0.05]. PFNA was also negatively associated with total BMD [*β* = −0.014, 95% CI = (−0.028, −0.001), *p* < 0.05]. To adjust for the effects of other metals and PFASs on BMD, a multiple-metal linear regression model was constructed, including all PFASs and heavy metals. In this model, Mn remained negatively associated with lumbar spine BMD [*β* = −0.003, 95% CI = (−0.007, −0.001), *p* < 0.05] and total BMD [*β* = −0.004, 95% CI = (−0.005, −0.001), *p* < 0.05]. In the single-exposure linear regression models, Mn and PFNA showed significant associations with BMD, whereas in the multiple-metal linear regression models, the individual effects of the other eight pollutants on lumbar spine BMD and total BMD did not reach statistical significance. These findings are provided in Tables 3, 4.

**Table 3:** Results of linear regression modeling for associations of the single-exposure with BMDs.

Single-exposure model^a^	Lumbar BMD		Total BMD	
	β	95%CI	β	95%CI
PFNA	−0.015	(−0.033, 0.004)	−0.014	(−0.028, −0.001)*
PFDA	−0.001	(−0.068, 0.067)	−0.008	(−0.056, 0.041)
PFUA	−0.056	(−0.160, 0.047)	−0.002	(−0.077, 0.073)
PFOA	−0.004	(−0.015, 0.008)	−0.007	(−0.015, 0.001)
PFOS	0.001	(−0.002, 0.005)	< 0.001	(−0.003, 0.002)
PFHxS	0.002	(−0.002, 0.007)	0.001	(−0.002, 0.004)
Cadmium	−0.045	(−0.097, 0.007)	−0.036	(−0.074, 0.001)
Lead	−0.002	(−0.027, 0.023)	−0.006	(−0.025, 0.012)
Manganese	−0.003	(−0.007, −0.001)*	−0.004	(−0.005, −0.001)*

CI, Confidence interval; *p < 0.05.

a Covariates adjusted included age, sex, body mass index, race, family income to poverty ratio, serum cotinine.

**Table 4:** Results of linear regression modeling for associations of the multiple-exposure with BMDs.

Multiple-exposure model^b^	Lumbar BMD			Total BMD		
	β	95%CI	VIF	β	95%CI	VIF
PFNA	−0.016	(−0.039, 0.007)	1.538	−0.013	(−0.029, 0.004)	1.844
PFDA	0.075	(−0.025, 0.176d)	2.342	0.045	(−0.028, 0.118)	2.302
PFUA	−0.088	(−0.222, 0.046)	1.831	0.017	(−0.080, 0.113)	1.711
PFOA	−0.009	(−0.024, 0.007)	2.068	−0.008	(−0.019, 0.003)	1.989
PFOS	0.002	(−0.002, 0.007)	2.019	0.000	(−0.004, 0.003)	2.126
PFHxS	0.003	(−0.003, 0.008)	1.479	0.002	(−0.001, 0.006)	1.409
Cadmium	−0.032	(−0.086, −0.021)	1.815	−0.028	(−0.066, 0.011)	1.876
Lead	0.003	(−0.023, 0.029)	1.212	−0.002	(−0.021, 0.017)	1.241
Manganese	−0.004	(−0.007, − 0.001) *	1.183	−0.003	(−0.005, − 0.001) *	1.158

CI, Confidence interval; *p < 0.05.

b Covariates adjusted included age, sex, body mass index, race, family income to poverty ratio, serum cotinine, PFNA, PFDA, PFUA, PFOA, PFOS, PFHxS, Cadmium, Lead, and Manganese.

In the linear regression analysis, factors including age, race, PIR, BMI, 25(OH)D, serum cotinine, PFOA, PFOS, PFNA, PFUA, PFDA, PFHxS, cadmium, lead, and manganese were incorporated to investigate their impact on lumbar spine BMD. The results showed that age, race, BMI and blood manganese were significant influencing factors of lumbar spine BMD in males (*p* < 0.05). Lumbar spine BMD increased by 0.003 g/cm^2^ for every 1 kg/m^2^ increase in BMI [95% CI = (0.001, 0.006), *p* = 0.008]. Additionally, lumbar spine BMD decreased by 0.007 g/cm^2^ [95% CI = (−0.011, −0.002), *p* = 0.009] with an increase of 1 μg/L in blood manganese. Among females, age, race, and BMI were identified as significant influencing factors of lumbar spine BMD (all *p* < 0.05). Specifically, lumbar spine BMD increased by 0.003 g/cm^2^ for every 1 kg/m^2^ increase in BMI [95% CI = (0.000, 0.005), *p* = 0.030], as shown in Tables 5, 6.

**Table 5:** Analysis of influencing factors of lumbar spine BMD in male.

Variable	β	95%*CI*	*t*	VIF	*p*
Age	0.039	(0.032, 0.046)	10.991	1.200	<0.001
Race	0.017	(0.005, 0.028)	2.896	1.118	0.004
PIR	0.005	(−0.005, 0.016)	1.005	1.162	0.315
BMI	0.003	(0.001, 0.006)	2.688	1.101	0.008
25(OH)D (nmol/L)	<0.001	(−0.001, 0.001)	0.515	1.131	0.607
Serum cotinine (ng/mL)	<0.001	(0.000, 0.000)	−0.341	1.781	0.733
PFNA (ng/mL)	−0.019	(−0.054, 0.016)	−1.087	1.844	0.278
PFDA (ng/mL)	0.078	(−0.049, 0.205)	1.208	2.302	0.228
PFUA (ng/mL)	0.012	(−0.195, 0.220)	0.117	1.711	0.907
PF0A (ng/mL)	−0.012	(−0.032, 0.008)	−1.150	1.989	0.251
PFOS (ng/ml)	0.003	(−0.003, 0.010)	1.061	2.126	0.290
PFHxS (ng/mL)	0.002	(−0.004, 0.009)	0.649	1.409	0.517
Cadmium (μg/L)	−0.024	(−0.085, 0.038)	−0.754	1.876	0.452
Lead (μg/dL)	<0.001	(−0.034, 0.034)	0.006	1.241	0.995
Manganese (μg/L)	−0.007	(−0.011, −0.002)	−2.648	1.158	0.009

**Table 6:** Analysis of influencing factors of lumbar spine BMD in female.

Variable	β	95%*CI*	*t*	VIF	*p*
Age	0.016	(0.010, 0.023)	4.816	1.184	<0.001
Race	0.013	(0.002, 0.024)	2.403	1.176	0.017
PIR	−0.003	(−0.012, 0.007)	−0.508	1.240	0.612
BMI	0.003	(0.000, 0.005)	2.177	1.082	0.030
25(OH)D (nmol/L)	<0.001	(0.000, 0.001)	1.031	1.164	0.303
Serum cotinine (ng/mL)	<0.001	(0.000, 0.000)	0.091	1.982	0.927
PFNA (ng/mL)	−0.018	(−0.047, 0.012)	−1.195	1.369	0.233
PFDA (ng/mL)	0.132	(−0.039, 0.303)	1.521	2.747	0.129
PFUA (ng/mL)	−0.149	(−0.338, 0.039)	−1.558	2.415	0.120
PF0A (ng/mL)	−0.010	(−0.035, 0.016)	−0.736	2.239	0.462
PFOS (ng/mL)	−0.002	(−0.009, 0.005)	−0.534	1.940	0.594
PFHxS (ng/mL)	0.003	(−0.005,0.011)	0.693	1.679	0.489
Cadmium (μg/L)	−0.045	(−0.168, 0.078)	−0.725	2.150	0.469
Lead (μg/dL)	−0.007	(−0.048, 0.035)	−0.331	1.099	0.741
Manganese (μg/L)	−0.002	(−0.006, 0.002)	−1.065	1.070	0.288

In the linear regression analysis, factors including age, Race, PIR, BMI, 25(OH)D, serum cotinine, PFOA, PFOS, PFNA, PFUA, PFDA, PFHxS, cadmium, lead, and manganese were included to further explore the influencing factors of total BMD. The results revealed that in the male group, age, race, BMI, PFOA, and blood manganese were significant influencing factors of total BMD (*p* < 0.05). Specifically, total BMD increased by 0.035 g/cm^2^ with every increase of 1 year in age [95% CI = (0.030, 0.040), *p* < 0.001], and increased by 0.003 g/cm^2^ for every 1 kg/m^2^ increase in BMI [95% CI = (0.002, 0.005), *p* < 0.001]. Conversely, for every 1 ng/mL increase of PFOA, total BMD decreased by 0.016 g/cm^2^ [95% CI = (−0.031, −0.001), *p* = 0.040], and for every 1 μg/L increase of blood manganese, total BMD decreased by 0.005 g/cm^2^ [95% CI = (−0.009, −0.002), *p* = 0.004]. In the female group, age, BMI, and PFNA were identified as significant influencing factors of total BMD (*p* < 0.05). The total BMD increased by 0.016 g/cm^2^ for every increase of 1 year in age [95% CI = (0.012, 0.021), *p* < 0.001], and increased by 0.005 g/cm^2^ for every 1 kg/m^2^ increase in BMI [95% CI = (0.003, 0.006), *p* < 0.001]. For every 1 ng/mL increase of PFNA, total BMD decreased by 0.019 g/cm^2^ [95% CI = (−0.039, 0.000), *p* = 0.048], as illustrated in Tables 7, 8.

**Table 7:** Analysis of influencing factors of total BMD in male.

Variable	β	95%*CI*	*t*	VIF	*p*
Age	0.035	(0.030, 0.040)	13.032	1.200	<0.001
Race	0.013	(0.004, 0.021)	2.940	1.118	0.004
PIR	0.001	(−0.007, 0.009)	0.244	1.162	0.807
BMI	0.003	(0.002, 0.005)	3.735	1.101	<0.001
25(OH)D (nmol/L)	<0.001	(0.000,0.001)	1.173	1.131	0.242
Serum cotinine (ng/mL)	<0.001	(0.000, 0.000)	0.007	1.781	0.994
PFNA (ng/mL)	−0.009	(−0.036, 0.017)	−0.705	1.844	0.481
PFDA (ng/mL)	0.078	(−0.018, 0.174)	1.605	2.302	0.110
PFUA (ng/mL)	0.143	(−0.013, 0.300)	1.798	1.711	0.073
PF0A (ng/mL)	−0.016	(−0.031, −0.001)	−2.059	1.989	0.040
PFOS (ng/mL)	−0.002	(−0.006, 0.003)	−0.632	2.126	0.528
PFHxS (ng/mL)	0.003	(−0.003, 0.008)	0.978	1.409	0.329
Cadmium (μg/L)	−0.030	(−0.077, 0.016)	−1.273	1.876	0.204
Lead (μg/dL)	0.003	(−0.022, 0.029)	0.246	1.241	0.806
Manganese (μg/L)	−0.005	(−0.009, −0.002)	−2.875	1.158	0.004

**Table 8:** Analysis of influencing factors of total BMD in female.

Variable	β	95%*CI*	*t*	VIF	*p*
Age	0.016	(0.012, 0.021)	7.192	1.184	<0.001
Race	0.007	(0.000, 0.014)	1.909	1.176	0.057
PIR	−0.004	(−0.010, 0.003)	−1.150	1.240	0.251
BMI	0.005	(0.003, 0.006)	6.241	1.082	<0.001
25(OH)D (nmol/L)	<0.001	(0.000, 0.001)	1.402	1.164	0.162
Serum cotinine (ng/mL)	<0.001	(0.000, 0.000)	−0.279	1.982	0.780
PFNA (ng/mL)	−0.019	(−0.039, 0.000)	−1.988	1.369	0.048
PFDA (ng/mL)	0.035	(−0.078, 0.147)	0.607	2.747	0.544
PFUA (ng/mL)	−0.029	(−0.153, 0.096)	−0.452	2.415	0.651
PF0A (ng/mL)	−0.001	(−0.018, 0.016)	−0.149	2.239	0.881
PFOS (ng/mL)	−0.002	(−0.007, 0.002)	−1.407	1.940	0.296
PFHxS (ng/mL)	0.003	(−0.002, 0.008)	1.058	1.679	0.291
Cadmium (μg/L)	−0.012	(−0.093,0.069)	−0.286	2.150	0.775
Lead (μg/dL)	−0.026	(−0.053, 0.002)	−1.838	1.099	0.067
Manganese (μg/L)	−0.002	(−0.005, 0.000)	−1.766	1.070	0.079

### 3.4 BKMR analysis

**Figure 2:**
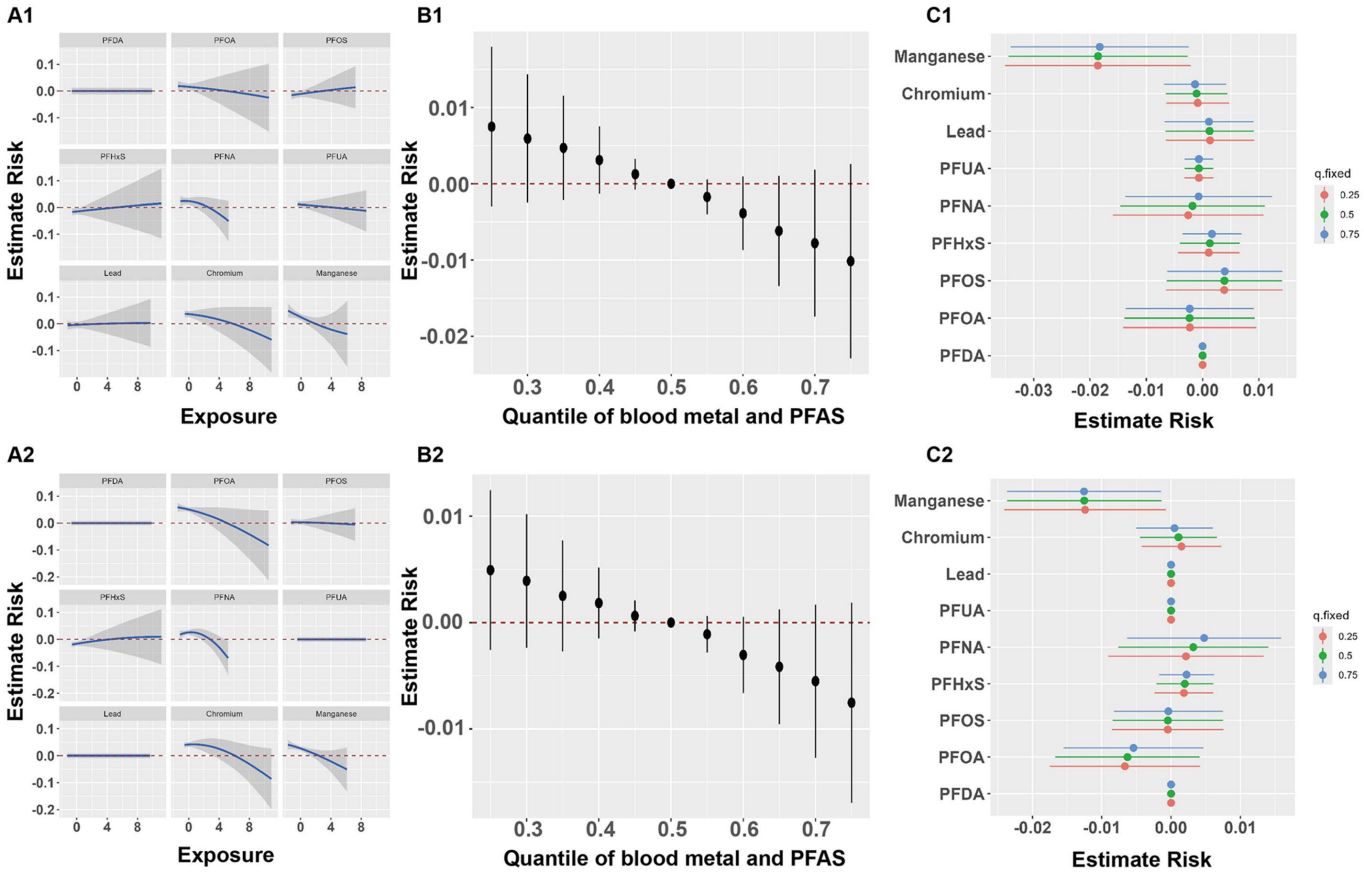
Joint effect of the metals and PFASs on BMD. Joint effect of the metals and PFASs on lumbar BMD **(A1–C1)** and total BMD **(A2–C2)**. **(A1,A2)** Effect of single metal and PFAS on BMD when other chemicals are fixed as the median (based on BKMR). **(B1,B2)** Overall effect of metals and PFASs mixture at particular percentiles compared with their 50th percentile (based on BKMR); Dots represent estimated values of the overall effect, and vertical lines represent the 95% confidence interval. **(C1,C2)** Effect of single metal and PFAS variable at the 25–75th percentile on the BMD when other exposure was fixed at the 25, 50, and 75th percentiles (based on BKMR).

Based on the findings from the BKMR model, overall associations were identified between multiple heavy metals, PFASs, and both lumbar spine and total BMD, with higher exposure levels showing a negative association with lumbar spine and total BMD (B1 and B2 in Figure 2). Results of PIPs suggested blood manganese contributed the most to the association between lumbar spine BMD and total BMD (Table 9). The exposure-response relationships between each of the three heavy metals, six PFASs, and BMD were also investigated, with exposure levels of the other eight pollutants set at their representative 50th percentiles. Nonlinear effects of Mn on both lumbar spine and total BMD were observed (A1 and A2 in Figure 2). The associations between individual perfluorinated compounds or heavy metals on the lumbar spine and total BMD are depicted in C1 and C2 in Figure 2, with other compounds set at the 25, 50, and 75th percentiles. A consistent negative correlation between blood Mn and both lumbar spine and total BMD was observed.

**Table 9:** Results of PIPs.

Pollutant	Lumbar BMD	Total BMD
PIP		
PFDA	<0.001	<0.001
PFOA	0.032	0.078
PFOS	0.018	0.002
PFHxS	0.052	0.009
PFNA	0.212	0.385
PFUA	0.013	<0.001
Lead	0.017	<0.001
Cadmium	0.764	0.070
Manganese	0/343	0.145

## 4 Discussion

Our study identified statistically significant differences in the distribution of total BMD and lumbar spine BMD between males and females. Sex hormones are one of the determinants of BMD and are thought to play an important role in increasing BMD and promoting bone maturation during adolescence. The significant rise in sex hormone levels during this period leads to differences in bone development between boys and girls as a result of these hormonal variations (29).

In this study, the concentrations of the majority of PFASs were lower than those reported in previous studies. For example, in American children aged 7.9 ± 0.8 years, the concentrations of PFOA, PFOS, PFDA, PFHxS, and PFNA were 4.4 ng/mL, 6.4 ng/mL, 0.3 ng/mL, 1.9 ng/mL, and 1.5 ng/mL, respectively (30). A study in the United Kingdom reported that the concentrations of PFOA, PFOS, PFHxS, and PFNA in 17-year-old females were 4.08 ± 1.83 ng/mL, 21.92 ± 10.72 ng/mL, 2.59 ± 5.36 ng/mL, and 0.56 ± 0.22 ng/mL, respectively (31). The variation in PFAS levels across different populations may be due to differing levels of industrialization in regions, as well as lifestyle and dietary factors.

Several epidemiological studies have discussed the association between exposure to PFASs and bone health (32–36). For example, an assessment by Xiong (34) of the correlation between serum levels of four PFASs (i.e., PFOA, PFOS, PFHxS, and PFNA) and BMDs of the total femur, femoral neck, and lumbar spine found that elevated serum levels of PFASs were associated with diminished BMD. The negative impact of PFASs on BMD was more pronounced in individuals who were overweight/obese. In a study conducted in the United States involving 48 obese children aged 8–12 years, a negative correlation between PFNA and BMD was observed, suggesting that exposure to PFASs in obese children may play a role in poor bone and cardiovascular health conditions (35). Another study (36) involving 1,260 Chinese adults found that exposure to higher concentrations of PFASs was significantly correlated with reduced BMD and an increased prevalence of osteoporosis; moreover, the study discovered that sex and age might act as modulating factors in the association between PFASs and bone health, with females and individuals under 60 years old possibly being more susceptible. Our study findings are similar to some of the previous research results. Specifically, we observed a correlation between higher concentrations of PFOA and lower BMD levels in the male group, which was consistent with the findings reported by Carwile et al. (32). Also, we found that a higher concentration of PFNA was associated with lower BMD levels in the female group, which is partially consistent with the findings of Blomberg (37), who found that the association between PFNA and BMD was significantly stronger in men than in women. Nonetheless, we did not find an association between blood PFNA concentration and BMD in men. In addition, our study did not confirm the associations between PFOS, PFDA, and PFHxS with BMD observed in previous studies (34, 35).

The mechanism of the impact of exposure to PFASs on bone health is not clear, but several possible biological pathways have been proposed in related studies. Firstly, PFASs can activate peroxisome proliferator-activated receptor-*γ*, disrupting the formation of osteoblasts and thereby affecting bone health (38). Secondly, as a type of EDCs, PFASs can cause changes in sex hormones and thyroid hormones that maintain bone health (39, 40), thereby compromising bone health. Lastly, PFASs can induce an imbalance between pro-inflammatory and anti-inflammatory systems, affecting osteoblast activity (41–43).

Current research on the association between heavy metals exposure and BMD in children and adolescents is limited. We found that blood manganese levels were higher in girls (12.310 ± 3.941 μg/L) than in boys (9.978 ± 3.166 μg/L), but our observations diverged from those reported by Liu et al. (44). A negative correlation between the blood manganese level and BMD was noted in males, whereas no discernible association was evident between the blood manganese concentrations and BMD in females. However, the association was not confirmed between lead, cadmium, and bone density observed in previous studies (45–47). The heterogeneity between our study and previous studies may stem from the sample size of several studies, variation in exposure levels, and different biological specimens. Studies on the correlation between exposure to heavy metals and BMD in children and adolescents are limited, and forthcoming large-scale prospective studies are required to validate our findings.

In our study, we found that co-exposure to heavy metals and PFASs was negatively associated with BMD. Both linear regression and multipollutant effect analysis indicated that Mn exposure may contribute to reduced BMD. Manganese is essential for the synthesis of glycosaminoglycans in the bone matrix and serves as a cofactor for certain enzymes involved in bone metabolism. The specific mechanisms by which manganese affects BMD are not fully understood, but several potential mechanisms have been proposed in related research. Firstly, manganese superoxide dismutase and pyruvate carboxylase play significant roles in bone metabolism, and as a cofactor for these enzymes, excessive manganese may lead to abnormal enzyme activity (48, 49). Secondly, exposure to manganese may affect the regulation of intracellular calcium content and ERK signaling, thereby inhibiting bone formation and development (50). Thirdly, manganese concentrations might alter the levels of other metal elements in bones, such as increased levels of iron and zinc and decreased copper, thereby affecting the generation of cytokines in bone metabolism and impacting bone health (51, 52). Lastly, excessive exposure to manganese can increase the generation of reactive oxygen species and exacerbate oxidative stress, affecting the activity of osteoblasts (53). Therefore, excessive exposure to Mn can potentially cause enzymatic abnormalities that impact bone mass. To date, few studies have explored the combined effects of heavy metals and PFASs on BMD, highlighting the need for further research to elucidate the molecular mechanisms underlying the joint effects of these environmental pollutants.

This study possesses several strengths. First of all, it leverages data from the four most recent NHANES cycles, benefiting from the rigorous quality control procedures implemented by NHANES. Secondly, the substantial sample size and the implementation of stratified analysis enhance the ability to identify potentially vulnerable subgroups based on sex and age. In addition, this study underscores that exposure to PFASs and heavy metals may exert more pronounced adverse effects on the health of susceptible populations, with potentially significant implications for public health. Nevertheless, it is essential to acknowledge the limitations inherent in this study. Firstly, owing to its cross-sectional design, we are precluded from establishing a definitive causal relationship between the exposure to PFASs, heavy metals and BMD; more prospective studies are needed to confirm whether increased concentrations of PFASs and heavy metals in the blood directly affect BMD. Therefore, the possibility of reverse causation cannot be excluded. Secondly, although serum concentrations of PFASs and heavy metals were measured, the duration of exposure to PFASs and heavy metals was not quantifiable in this study. Lastly, despite adjusting for various confounders, the study did not account for some potential confounders, including dietary intake, nutritional supplements, and medications. These factors might influence both bone health and the levels of PFASs and heavy metals.

## 5 Conclusion

We observed a correlation between elevated serum levels of PFASs and heavy metals and diminished BMD, with sex differences. Our findings suggested that exposure to PFASs and heavy metals may be associated with poor bone health. However, in light of the limitations inherent in the study, a prudent interpretation of the results is warranted. Further longitudinal studies, incorporating a comprehensive collection of confounders, are necessary to confirm these findings and provide a more accurate assessment of the associated risks.

## Data Availability

Publicly available datasets were analyzed in this study. This data can be found here: https://www.cdc.gov/nchs/nhanes/.

## Footnotes

^1^cdc.gov
